# Influenza A virus NS1 protein represses antiviral immune response by hijacking NF-κB to mediate transcription of type III IFN

**DOI:** 10.3389/fcimb.2022.998584

**Published:** 2022-09-15

**Authors:** Meng-Chang Lee, Cheng-Ping Yu, Xing-Hong Chen, Ming-Tsan Liu, Ji-Rong Yang, An-Yu Chen, Chih-Heng Huang

**Affiliations:** ^1^ School of Public Health, National Defense Medical Center, Taipei, Taiwan; ^2^ Graduate Institute of Life Sciences, National Defense Medical Center, Taipei, Taiwan; ^3^ Department of Pathology, Tri-Service General Hospital, National Defense Medical Center, Taipei, Taiwan; ^4^ Institute of Preventive Medicine, National Defense Medical Center, Taipei, Taiwan; ^5^ Center for Diagnostics and Vaccine Development, Centers for Disease Control, Taipei, Taiwan; ^6^ Graduate Institute of Medical Sciences, National Defense Medical Center, Taipei, Taiwan; ^7^ Department of Microbiology and Immunology, National Defense Medical Center, Taipei, Taiwan

**Keywords:** influenza, non-structural protein 1 (NS1), NF-κB, *IFNL1*, chromatin immunoprecipitation and sequencing (ChIP-seq)

## Abstract

**Background:**

Non-structural protein 1 (NS1), one of the viral proteins of influenza A viruses (IAVs), plays a crucial role in evading host antiviral immune response. It is known that the IAV NS1 protein regulates the antiviral genes response mainly through several different molecular mechanisms in cytoplasm. Current evidence suggests that NS1 represses the transcription of *IFNB1* gene by inhibiting the recruitment of Pol II to its exons and promoters in infected cells. However, IAV NS1 whether can utilize a common mechanism to antagonize antiviral response by interacting with cellular DNA and immune-related transcription factors in the nucleus, is not yet clear.

**Methods:**

Chromatin immunoprecipitation and sequencing (ChIP-seq) was used to determine genome-wide transcriptional DNA-binding sites for NS1 and NF-κB in viral infection. Next, we used ChIP-reChIP, luciferase reporter assay and secreted embryonic alkaline phosphatase (SEAP) assay to provide information on the dynamic binding of NS1 and NF-κB to chromatin. RNA sequencing (RNA-seq) transcriptomic analyses were used to explore the critical role of NS1 and NF-κB in IAV infection as well as the detailed processes governing host antiviral response.

**Results:**

Herein, NS1 was found to co-localize with NF-κB using ChIP-seq. ChIP-reChIP and luciferase reporter assay confirmed the co-localization of NS1 and NF-κB at type III IFN genes, such as *IFNL1*, *IFNL2*, and *IFNL3*. We discovered that NS1 disturbed binding manners of NF-κB to inhibit *IFNL1* expression. NS1 hijacked NF-κB from a typical *IFNL1* promoter to the exon-intron region of *IFNL1* and decreased the enrichment of RNA polymerase II and H3K27ac, a chromatin accessibility marker, in the promoter region of *IFNL1* during IAV infection, consequently reducing *IFNL1* gene expression. NS1 deletion enhanced the enrichment of RNA polymerase II at the *IFNL1* promoter and promoted its expression.

**Conclusion:**

Overall, NS1 hijacked NF-κB to prevent its interaction with the *IFNL1* promoter and restricted the open chromatin architecture of the promoter, thereby abating antiviral gene expression.

## Introduction

Influenza A virus (IAV) is one of the most common causes of hospitalization due to pneumonia and mortality each year (Flerlage et al., 2021; Lafond et al., 2021). The influenza (Flu) virus is associated with a shorter incubation time (ranging from 1–4 days) and a more rapid and acute onset of pneumonia symptoms (Taubenberger and Morens, 2008; Paules and Subbarao, 2017). Clinically, the histopathology of severe flu pneumonia is characterized by necrotizing bronchitis and bronchiolitis, hyperemia of the alveolar wall, hemorrhage, inflammatory cell infiltration, hyaline membrane formation, and pulmonary edema. Moreover, small vessel and capillary thrombosis and diffuse alveolar damage cause coagulation disorders and severe hypoxemia (Davison et al., 1973; Taubenberger and Morens, 2008; Short et al., 2014; Yang and Tang, 2016). Exaggerated alveolar expression of inflammatory mediators and proinflammatory cytokines are the main causes of severe lung injury during viral infection (Cheung et al., 2002; de Jong et al., 2006; Baskin et al., 2009). Based on prior evidence, a finely tuned type III and type I IFN-mediated antiviral response plays an essential role in balancing immunity for antiviral protection and host damage (Crotta et al., 2013; Davidson et al., 2014; Galani et al., 2017; Andreakos et al., 2019). Acute respiratory distress syndrome (ARDS) is the main reason for severe disease outcomes following flu and heart failure is a related consequence of this disease (Arabi et al., 2007; Garg et al., 2015; Sellers et al., 2017). However, the molecular risk factors that cause disease progression in mild to severe flu patients are unclear. Recently, a small cohort study suggested that IFN-λ1 and type I IFN were swiftly induced earlier at higher levels and independently of disease severity in noncritically and critically ill patients with flu. In addition, type I IFN levels rapidly declined on day 3 after hospital admission, whereas IFN-λ1 was induced for a longer period. Notably, patients with flu who became critically ill had markedly reduced IFN-λ1 levels compared to noncritically ill patients on admission (Galani et al., 2021).

Flu viruses belong to the family, *Orthomyxoviridae*, and are classified into four types (influenza A, B, C, and D virus). However, only types A and B cause disease epidemics in people. Human flu A and B viruses have eight segments of negatively single-strand RNA genome that encode up to 17 and 11 viral proteins, respectively (Dubois et al., 2014), thereby facilitating viral infection and replication in the host (Dou et al., 2018; Krammer et al., 2018). The non-structural protein 1 (NS1) protein, which is encoded by genome segment 8, is a multifunctional virulence factor, the role of an additional factor in the infection (Hale et al., 2008; Cho et al., 2020). NS1 is also an IFN antagonist (García-Sastre, 2001; Geiss et al., 2002; Jia et al., 2010) that suppresses IFN-mediated antiviral response *via* multiple mechanisms (Ji et al., 2021). These mechanisms include (i) the inhibition of the retinoic-acid-inducible protein 1 (RIG-I)-mediated antiviral signaling pathway by preventing NF-κB (Wang et al., 2000), IFN-regulatory factor (IRF) 3 activation (Talon et al., 2000), and E3 ubiquitin ligase activity (TRIM25 and Riplet) (Gack et al., 2009; Rajsbaum et al., 2012); (ii) inactivation of vital components for the transcription of antiviral genes *via* binding to cellular RNAs, such as RIG-I (Mibayashi et al., 2007) and double-stranded RNA-dependent protein kinase R (PKR) (Onomoto et al., 2012); (iii) prevention of transcriptional machinery loading by binding cellular DNA (Anastasina et al., 2016); and (iv) inhibition of IFN pre-mRNA processing *via* CPSF30 binding (Nemeroff et al., 1998).

Generally, alveolar epithelial cell (AEC) apoptosis is an underlying mechanism of alveolar damage in murine and human models of adult respiratory distress syndrome (Guinee et al., 1997; Kitamura et al., 2001; Perl et al., 2007). In fact, in recent simulation studies, cell cultures were infected at high-multiplicity of infection (MOI) and the level of apoptotic cells started to increase at 16 hours post-infection (hpi) and reached their maximum of approximately 77% at 32 hpi (Frensing et al., 2016; Rüdiger et al., 2019). This study used human lung epithelial cells and infected cells with the flu virus at a high MOI to mimic human lung physiology during virus-induced apoptosis (Yeganeh et al., 2018).

Based on the NS1 characteristic of residence in the nucleus and binding to DNA, we employed the chromatin immunoprecipitation sequencing (ChIP-seq) technology to explore the whole genome binding sites of NS1 and NF-κB, respectively. Unexpectedly, over 70% overlapping rates were found to exist between NS1 and NF-κB binding sites, including binding peaks in the annotation of *IFNLs*. Notably, co-localization of NS1 and NF-κB was observed in the exon-intron region, and not at the specific promoter of *IFNL1.* Time course analyses using the ChIP assay revealed that NS1 hijacked NF-κB from its promoter to the exon-intron of *IFNL1*, induced a decline in the enrichment of RNA polymerase II (Pol II), and restricted open chromatin, ultimately abating the expression of the antiviral gene, *IFNL1*. Indeed, we demonstrate that the flu NS1 protein is associated with the intragenic region of *IFNL1*. Notably, this association was vital to the attenuation of type III IFNs by interfering with the intragenic recruitment of NF-κB in the early stage of high-MOI infection. Our findings provide insights into the novel role for viral proteins, such as IAV NS1, in the transcriptional control mechanisms involved in the complex virus-host interplay of viral immune antagonism and innate immune response at the early stages of infection in lung epithelial A549 cells.

## Methods

### Cells, viruses, and reagents

A549, A549-Dual™ (*In vivo*Gen), MDCK, and HEK 293T cells were cultured in Dulbecco’s modified Eagles medium (Gibco) supplemented with 1% 100x Glutamax (Gibco), 1% 100x Anti-Anti (Gibco), and 10% fetal bovine serum (Gibco) at 37°C in a 5% CO_2_ incubator. Influenza viruses A/WSN/33 (H1N1) were grown in MDCK cells, and virus titers were determined using the plaque assay.

### Secreted embryonic alkaline phosphatase assay

A549-Dual™ cells were seeded in 24-well plates at 2x10^5^ cells per well and grown to 90% confluence before infection. SEAP was measured by the QUANTI-Blue assay (*In vivo*Gen) on an Infinite 200 pro plate reader (TECAN), according to the manufacturer’s instructions.

### Chromatin immunoprecipitation and quantitative polymerase chain reaction

ChIP assay was performed according to the manufacturer’s instructions of the Magna ChIP™ A/G Chromatin Immunoprecipitation Kit (Merck, 17-10085). Materials, including 10X glycine, 10X PBS, cell lysis buffer, nuclear lysis buffer, Protease Inhibitor Cocktail II, RNase A, Proteinase K, protein A/G magnetic beads, dilution

buffer, low salt wash buffer, high salt wash buffer, LiCl wash buffer, TE buffer, elution buffer, and spin columns, were from Magna ChIP™ A/G Chromatin Immunoprecipitation Kit (Merck, 17-10085). Briefly, A549 cells were seeded in 150mm dish (Corning) and grown to around 90% confluency. After fixing cells with 1% formaldehyde (Merck) for 10 min and neutralizing with 1X glycine for 5 min, cells were washed with cold 1X PBS twice and harvested using cell scraper (Corning). Cells were centrifuged at 800 × g for 5 min at 4°C, followed by incubation in 500 μl cell lysis buffer for 15 min on ice and performed a quick vortex for 3 sec at half maximum speed to mix every 5 min. Cell pellets were collected *via* centrifugation at 800 × g for 5 min at 4°C and re-suspended with 500 μl of nuclear lysis buffer. DNA was fragmented using Q700 Sonicator (Qsonica), followed by centrifugation at 10,000 × g for 10 min at 4°C. The supernatant was divided into 50-μl aliquots. For immunoprecipitation, each aliquot was added to 450 μl dilution buffer, 20 μl protein A/G magnetic beads, p65 (Cell Signaling Technology) or p50 (Cell Signaling Technology) or NS1 (GeneTex, GTX125990) or RNA polymerase II (Magna ChIP A/G MAGNA0014, 05-623B) or H3K27ac (Millipore, 07-360), and IgG antibody (Magna ChIP A/G MAGNA0014,12-371B) for overnight incubation at 4°C with agitation. A magnetic separator was used to adsorb bead-immune complexes in each tube, and the supernatant was removed. Bead-immune complexes were washed sequentially using 500 μl low salt-, high salt-, LiCl-immune complex wash buffer, and TE buffer. Elution buffer with RNase A was added to the bead-immune complexes and incubated at 37°C for 30 min. Proteinase K was then added and incubated at 62°C for 2 h to elute the immune complexes from beads. The cross-linking reversal of the immune complexes was accomplished by incubation at 95°C for 10 min. DNA purification was conducted using commercial spin columns according to the manufacturer’s instructions. The eluted DNA aliquots in TE buffer were immediately stored at −20°C until needed. For the ChIP-qPCR assay, input DNA or antibody-enriched DNA fragments were amplified on the LightCycler^®^ 480 qPCR system (primer sequences in **Supplementary Table 1**). Each qPCR reaction was performed by the PrimeTime^®^ Gene Expression Master Mix (IDT 1055770), according to the manufacturer’s instructions. The results are presented as relative fold enrichment (ChIP/IgG).

### Chromatin immunoprecipitation-reChromatin immunoprecipitation and polymerase chain reaction

After primary ChIP, 10 mM DTT was used to elute complexes from the beads for 30 min at 37°C. The dilution buffer containing 1x Protease Inhibitor Cocktail II, protein A/G magnetic beads, and the subsequent antibodies was mixed with immune complexes on a rotary shaker at low speed and incubated overnight at 4°C. DNA purification and storage were carried out as described above. DNA was amplified with primers (**Supplementary Table 1**) *via* using a VELOCITY PCR kit (BIOLINE), according to the manufacturer’s instructions. Finally, PCR products were analyzed on a 2% agarose gel.

### ChIP-seq and data analysis

According to the manufacturer’s instructions, ChIP-seq was executed using HiSeq2500 or HiSeq4000. Trimmomatic software was used to trim lower-quality reads using default settings (Bolger et al., 2014). Reads were mapped to GRCh37/hg19 using Bowtie2 software (Langmead and Salzberg, 2012). Peak calling was achieved using Model-based Analysis of ChIP-seq data (MACS2) (Zhang et al., 2008; Feng et al., 2012). To further identify reliable peaks of p65, we loaded 2 packages of R programming language, rtracklayer and GenomicRanges, to compute overlapping peaks across replicates (Lawrence et al., 2009; Lawrence et al., 2013). First, we imported four replicates of p65 peaks files derived from MACS2 above in the R programming environment. Then, we compute overlapping of four replicates of p65 peaks whose criterion was set equal to or more than three times in four replicates of p65 peaks, to obtain final peaks. Peaks of p50 and NS1 were also finally determined using the same protocol above. Computation and visualization of peaks, shown in single-end, were achieved using Easeq (Lerdrup et al., 2016) or according to the usage of ChIP-seq data analysis in R (Park et al., 2017). To compare the discrepancy of genomic enrichment of p65 at the IFNL1 gene locus under Poly I:C and under influenza viruses challenge, we used Poly I:C ChIP-seq data from GSE91018 (Borghini et al., 2018). To compare the variation of genomic enrichment of RNA polymerase II under wild type influenza viruses and deletion of NS1 influenza viruses challenge, we used ChIP-seq data from GSE156060. Both of them were obtained from the National Center for Biotechnology Information. Motif analysis was performed using g:Profiler online tool (Raudvere et al., 2019).

### RNA extraction and real-time quantitative polymerase chain reaction

RNA extraction was conducted using TRIzol Reagent (Invitrogen) according to the manufacturer’s instructions. High-Capacity cDNA Reverse Transcription Kits (Applied Biosystems™, 4368814) were used to reverse transcribe RNA to cDNA. According to the manuals, TOOLS 2xSYBR qPCR Mix (FPT-BB05) or PrimeTime^®^ Gene Expression Master Mix (IDT 1055770) was used. The primer sequences are listed in **Supplementary Table S1**. RT-qPCR was performed on the LightCycler^®^ 480 qPCR system.

### RNA-sequencing and data analysis

RNA-sequencing data, including GSE147507 (Blanco-Melo et al., 2020), were obtained from NCBI. First, lower quality reads were trimmed using Trimmomatic software. Thereafter, reads were mapped to GRCh37/hg19 using HISAT2 software (Kim et al., 2015). The genes of mapped reads were counted using *featureCounts software* (Liao et al., 2014). Differential genes expression and Kyoto Encyclopedia of Genes and Genomes (KEGG) pathways were analyzed using iDEP online tool (Ge et al., 2018). Gene Set Enrichment Analysis (GSEA) software was used to compute and identify the enriched biological process (BP) and KEGG pathways (Subramanian et al., 2005). One can judge results by checking upward or downward peaks (positive or negative correlation with phenotype), normalized enrichment score (NES), nominal p-value, and false discovery rate (FDR) q-value in which nominal p-value <0.05 and FDR q-value <0.25 are criteria for significance. For more information, please refer to the reference (Subramanian et al., 2005).

### Immunofluorescence

A549 cells were seeded in 8-well chamber slides (Lab-TekII, Thermo) at 2x10^4^ cells per well and grown to 90% confluence before infection. The cells were washed with PBS and infected with WSN (H1N1) virus at an MOI of 5 for 2, 4, 6, 8, 10h. The WSN-infected cells were washed with PBS and fixed with 4% paraformaldehyde in PBS for 30 min at room temperature. The cells were then permeabilized with 0.3% TritonX-100 in PBS for 10 min and blocked with 5% skim milk in PBS for 1 h at RT. After blocking, the cells were incubated overnight with an NS1 antibody (GTX125990) at 4°C, and then incubated with Alexa Fluor 488-labeled goat anti-rabbit IgG. The cell nuclei were stained with 4’,6-diamidino-2-phenylindole (DAPI) followed by mounting with SlowFade Diamond antifade reagent (Thermo). Data were visualized using a Leica DMi8 fluorescence microscope.

### Luciferase reporter assay

Approximately 10000 HEK 293T cells were seeded in a 96-well plate and incubated for 16 to 18 h prior to plasmid transfection. Briefly, pMCS-Cypridina Luc Vector inserted with or without NF-κB and NS1 enriched locus, *IFNL1, IFNL2*, or *IFNL3* with red firefly luc control plasmid were co-transfected into HEK 293T cells for 48 h. The primers for subcloning are listed in **Supplementary Table S2**. The p65 (OriGene, RG220780), p50 (OriGene, SC127485), and NS1 (OriGene, VC102470) plasmids were co-transfected with each pMCS-Cypridina Luc Vector with or without the above-enriched locus and red firefly luc control plasmid into HEK293 cells as described above. Luciferase activity was measured 24 h post-transfection using the TECAN Infinite 200 reader and Pierce™ Cypridina-Firefly Luciferase Dual Assay Kit (Thermo Scientific) according to the manufacturer’s protocol.

### Statistical analysis

Data are presented as mean ± standard deviation. Statistical analyses were performed using Graphpad Prism 9 software. One-way ANOVA with Dunnett’s test were used for comparison among 3 or more groups. P < 0.05 was considered statistical significance (*P < 0.05, **P < 0.01, and ***P < 0.001).

## Results

### Co-localization of NF-κB and NS1 during IAV infection

**Figure 1:**
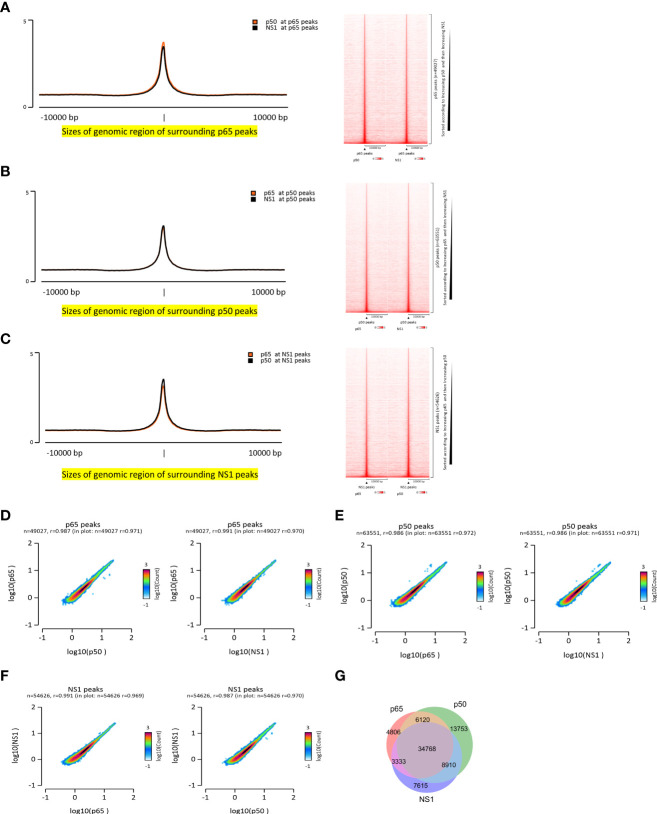
Co-localization of NF-κB and NS1 during IAV infection. **(A–C)** Enrichment signals of NF-κB and NS1 are depicted in the peak plot and heatmap aligned to NF-κB and NS1, respectively. **(D–F)** Pair correlation coefficient plot of the NF-κB- and NS1-enriched signals based on the NF-κB and NS1 peaks, respectively. **(G)** Venn plot of the NF-κB and NS1 intersection peaks.

To explore the whole genome binding sites of NF-κB (p65 and p50 subunits) and confirm whether NS1 binds to the host genome, ChIP-seq of p65, p50, and NS1 during IAV infection was conducted. A total of 49027, 63551, and 54626 binding peaks of p65, p50, and NS1, respectively, were found, with occupation rates of 24.9%, 22.2%, and 20.91% on the promoter (<=1 kb) (**Supplementary Figure 1**). Interestingly, the p50 and NS1 ChIP-seq average signals were enriched at the p65 peaks (**Figure 1A**). Similar phenomena were observed at p50 peaks, with significant p65 and NS1 enriched signals (**Figure 1B**), and NS1 peaks, with remarkable p65 and p50 enriched signals (**Figure 1C**). Pair correlation coefficient analysis of the p65, p50, and NS1 ChIP-seq results revealed a significant correlation between NF-κB and NS1 peak signals, indicating the co-localization of NF-κB and NS1 during infection (**Figures 1D-F**). A Venn diagram displaying the 34,768 intersection sites of p65, p50, and NS1 peaks is shown in **Figure 1G** and the physical distributions of these sites across the whole genome is depicted in **Supplementary Figure 1**. To confirm whether NS1 binds to NF-κB binding sites *in vivo*, we used an NF-κB SEAP reporter assay and A549-Dual cells that featured a secreted embryonic alkaline phosphatase (SEAP) reporter gene at NF-κB binding sites. NF-kB activity was significantly increased in IAV-infected or NS1-overexpressed A549-Dual Cells based on SEAP activity (**Supplementary Figure 2**). Together, these findings suggest that the nuclear localization of NS1 occurs at the NF-κB binding sites during IAV infection.

### Deletion of NS1 upregulates NF-κB signaling

**Figure 2:**
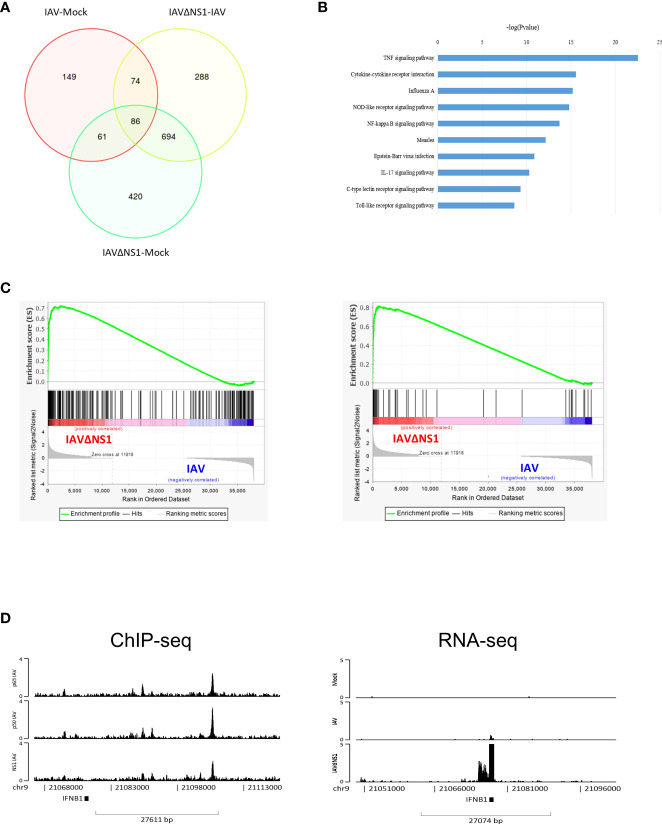
Deletion of the NS1-triggered upregulation of NF-κB signaling. **(A)** Venn plot of differentially expressed genes (DEGs) among the mock, IAV, and IAVΔNS1 groups. **(B)** Bar chart of the selectively enriched pathways for DEGs between and IAV. **(C)** Gene set enrichment analysis of the comparison of IAVΔNS1 and IAV. **(D)** Genomic binding of NF-κB and NS1 at the *IFNB1* gene loci derived from the ChIP-seq data. IFNB1 mRNA abundance derived from the RNA-seq data for the mock, IAV, and IAVΔNS1 groups.

To further understand the role of NS1 in flu infection, we compared the RNAseq of wild-type WSN and the deletion of NS1 on IAV-induced (IAVΔNS1) host responses. Some differentially expressed genes (DEGs) were found among mock, IAV, and IAVΔNS1 as depicted in **Supplementary Figure S3A**. The Venn plot showed that the DEG induced by IAVΔNS1 was substantially different from that of wild-type IAV (**Figure 2A**). The top five upregulated enrichment pathways for DEGs of IAVΔNS1 compared with IAV were the TNF signaling pathway, cytokine-cytokine receptor interaction, IAV, NOD-like receptor signaling pathway, and NF-κB signaling pathway (**Figure 2B**). To further explore differential expression of gene sets in host between IAVΔNS1 and wild-type IAV conditions, we used GSEA software to analyze the role of NS1 in the process of infection. Gene set enrichment analysis (GSEA) also revealed that the lack of NS1 reinforced *Toll*-*like receptor (TLRs) signaling pathway* (normalized enrichment score, NES: 1.86; nominal p-value: 0.0; False discovery rate, FDR q-value: 1.88E-4) and *RIG-I like receptor signaling pathway* (NES: 1.92; nominal p-value: 0.0; FDR q-value:5.64E-4) (**Supplementary Figure 3B**), which are well-known triggers of the NF-kappa B signaling pathway, as illustrated in the KEGG pathway diagram (**Supplementary Figure 3C**). The lack of NS1 not only strengthened the response to type I interferon (NES: 2.28; nominal p-value: 0.0; FDR q-value: 0.0) and emphasized the response to interferon-gamma (NES: 2.26; nominal p-value: 0.0; FDR q-value: 0.0) (**Supplementary Figure 3D**), but also enhanced the defense response to the virus (NES: 2.19; nominal p-value: 0.0; FDR q-value: 0.0) and promoted negative regulation of viral genome replication (NES: 2.08; nominal p-value: 0.0; FDR q-value: 0.0) (**Figure 2C**), revealing the role of NS1 inhibition in host immune responses. ChIP-seq revealed the co-localization of NF-κB and NS1 at the region annotated to *IFNB1* and RNA-seq revealed the upregulation of *IFNB1* upon NS1 deletion (**Figure 2D**). When treated with the NF-κB inhibitor, Bay11, *IFNB1* was downregulated in cells (**Supplementary Figure 4**), implicating the competitive roles of NF-κB and NS1 in regulating antiviral genes *via* DNA-mediated protein interaction.

### Co-localization of NF-κB and NS1 at type III interferons in IAV-infected A549 cells

**Figure 3:**
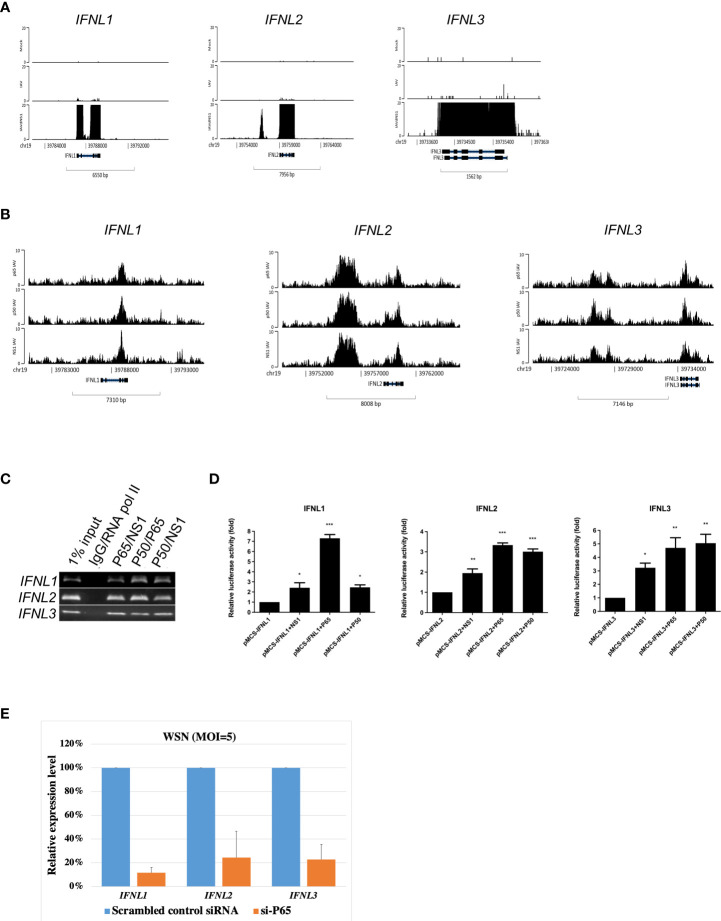
Co-localization of NF-κB and NS1 at type III interferons in IAV-infected A549 cells. **(A)** Genomic abundance of *IFNL1, IFNL2, IFNL3* expression derived from RNA-seq data upon mock, IAV, and IAVΔNS1 groups. **(B)** Genomic binding of NF-κB and NS1 at the *IFNL1, IFNL2*, and *IFNL3* gene loci derived from the ChIP-seq data. **(C)** ChIP-reChIP PCR results were displayed using agarose gel. **(D)** Relative luciferase activity when the reporter plasmid, enriched loci individually annotated to *IFNL1, IFNL2*, and *IFNL3*, was co-transfected with the control or NF-κB/NS1 plasmid, respectively. **(E)** A549 cells were transfected with scramble, control siRNA or NF-κB siRNA (si-P65; 30 pmol) for 48 hours, then were infected with WSN (MOI=5) for 8 hours. Relative expression levels of *IFNL1, IFNL2*, and *IFNL3* mRNA were quantified by qRT-PCR. GAPDH mRNA was presented as internal controls for mRNA loading. To compare the relative expression levels of three different type III IFNs, values were normalized to that of scrambled siRNA treated cells. Each experiment was performed in triplicate. Data are presented as mean ± SD, n = 3. *P  < 0.05; **P  < 0.01, and ***P  < 0.001 vs. control.

Besides *IFNB1*, type III IFN genes, such as *IFNL1, IFNL2*, and *IFNL3*, which are upregulated in the enrichment pathways after *NS1* deletion (**Figure 3A**), were also the targets of NF-κB and NS1 co-localization (**Figure 3B**). To further confirm this discovery, a ChIP-reChIP assay was used to validate NF-κB and NS1 interaction at *IFNLs.* NF-κB and NS1 were found to co-localize at *IFNL1, IFNL2*, and *IFNL3*, respectively (**Figure 3C**). NF-κB and NS1 displayed reporting activity of three loci annotated to *IFNL1, IFNL2*, and *IFNL3*, respectively (**Figure 3D**). Based on the results of RNA-seq and ChIP-seq, we hypothesized that the co-localization of NF-κB and NS1 could have important implications for the target gene during infection with IAV. We next used an siRNA approach to determine the effect of NF-κB knockdown on the expression of *IFNL1*, *IFNL2*, and *IFNL3* genes in IAV-infected A549 cells. The results showed that the inhibition of NF-κB led to the reduction of type III IFNs expression (**Figure 3E**). Together, these results suggest that IAV NS1 can act in the nucleus to alter the expression of type III IFNs upon IAV infection.

### NS1 hijacks NF-κB from *the IFNL1* promoter to the exon-intron region and attenuates the enrichment of RNA polymerase II and H3K27ac to inhibit *IFNL1* expression

**Figure 4:**
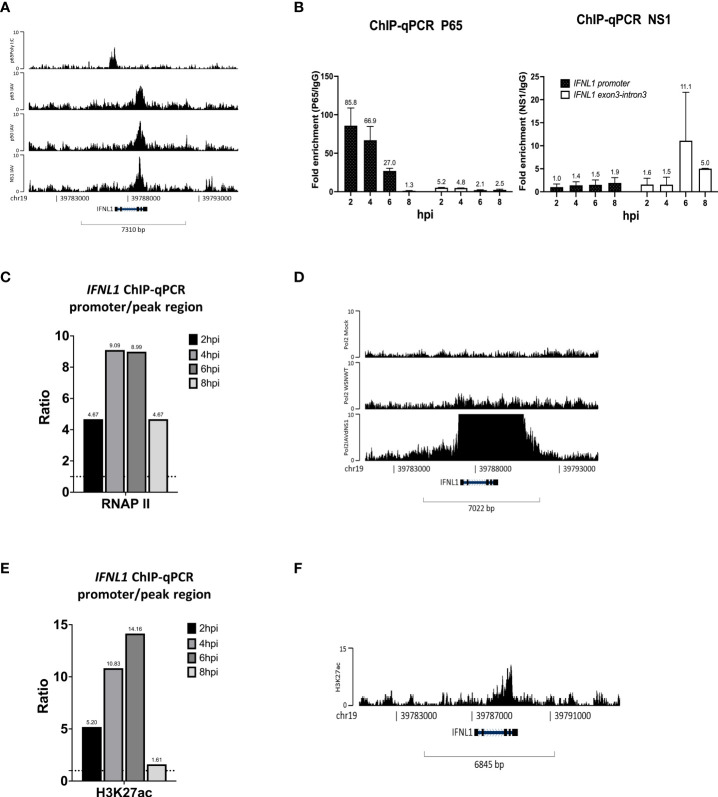
NS1 hijacks NF-κB from the *IFNL1* promoter to the exon-intron region and attenuates the enrichment of RNA polymerase II and H3K27ac to inhibit *IFNL1* expression. **(A)** Genomic binding of p65 at the *IFNL1* gene locus derived from our ChIP-seq data and GSE91018. **(B–D)** Relative fold enrichment ratios of p65, NS1 **(B)**, RNA polymerase II **(C)**, and H3K27ac **(D)** at the promoter and exon-intron region of the *IFNL1* gene locus based on time course analysis. **(E)** Genomic enrichment of RNA polymerase II at the *IFNL1* gene locus derived from the ChIP-seq data (GSE156060) for the mock, IAV, and IAVΔNS1 groups. **(F)** Genomic binding of H3K27ac at the *IFNL1* gene loci derived from the ChIP-seq data.

Unexpectedly, at 8 hpi, the co-localization of NF-κB and NS1 was identified at *the IFNL1* exon-intron region, and not on a specific promoter, compared to the co-localization region with p65 binding sites at the promoter upon Poly I:C stimulus, which mimicked double-strand RNA stimulation (**Figure 4A**). Accordingly, we postulated that the NF-κB binding manner was affected, or to be exact, hijacked by NS1. To confirm this hypothesis, we conducted time course experiments of IAV infection, followed by a ChIP assay against p65 and IgG antibodies and specific primers for the *IFNL1* promoter and co-localization region of *IFNL1* exon-intron using qPCR. In addition, we also determined the kinetics of *IFNLs* gene expression for the indicated time in the early stages of infection (**Supplementary Figure 5**). **Figure 4B** shows that the relative p65 fold enrichment ratios of the *IFNL1* promoter to the co-localization region plummeted between 6 hpi and 8 hpi, indicating displacement of p65 from the promoter to the co-localization region of NF-κB and NS1 owing to IAV. Thereafter, an immunofluorescence assay (IFA) was conducted and NS1 was identified to substantially accumulate at the nucleus after 6 hpi (**Supplementary Figure 6**). The relative RNA polymerase II fold enrichment of the *IFNL1* promoter to the co-localization region also declined after reaching its peak at 4 hpi (**Figure 4C**). Without NS1, RNA polymerase II enrichment was sharply enhanced at *IFNL1* (**Figure 4D**) when infected with IAV, aligning with the results presented in **Figure 3A**. We also observed that the enrichment of H3K27ac, a marker of open chromatin, at the *IFNL1* promoter abated after 6 hpi (**Figure 4E**), suggesting that this region might be assembled into closed chromatin after flu infection. The ChIP-seq data revealed disappearing enrichment of H3K27ac at the *IFNL1* promoter but its accumulation at the co-localization region of NF-κB and NS1 (**Figure 4F**). Altogether, these results indicate that NS1 forced the displacement of NF-κB from the promoter, reduced the enrichment of RNA polymerase II, and modified the chromatin structure, ultimately inhibiting *IFNL1* expression (**Supplementary Figure 5**).

### Regulatory motif analysis of the co-localization of NF-κB and NS1

**Figure 5:**
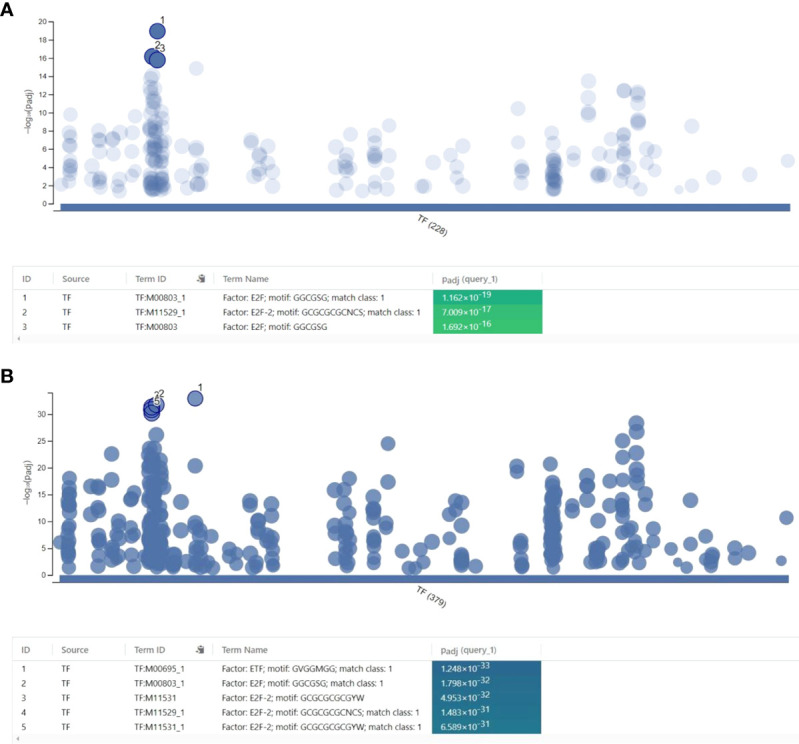
Regulatory motif analysis of the co-localization of NF-κB and NS1. **(A)** Motif analysis of the co-localization of NF-κB and NS1 at promoter regions less than 3000 base pairs. **(B)** Motif analysis of the co-localization of NF-κB and the NS1 regions.

As NS1 is well-known for interacting with RNA and proteins to accomplish its role as a nuclear protein during IAV infection, genome-wide protein-RNA interactome studies revealed that NS1 binds to a wide range of dsRNA sequences *via* its N-terminal RNA-binding domain. However, no sequence specificity was identified (Zhang et al., 2018). NS1 can bind to synthetic dsDNA in a non-specific sequence manner to inhibit the transcriptional reaction *in vitro* and prevent the recruitment of RNA Pol II to exon and promoter regions of the target gene in IAV infected cells (Anastasina et al., 2016). Here, we found that NS1 colocalized with the transcription factor NF-κB bind at chromatin was related to affect gene transcription. However, owing to the co-binding of viral protein and transcription factor at the same regulatory DNA elements, the extent to which this process is relevant to immune gene regulation during IAV infection remains unknown. Only few studies have indicated that NS1 can interact with specific DNA sequences at the promoter region, in addition to cellular RNA (Marc et al., 2012; Ji et al., 2021). We proceeded to determine whether NS1 and NF-κB co-localization might disrupt the activity of other transcription factors that regulate the transcription of immune response genes during infection; hence, motif enrichment analysis (MEA) of the NF-κB and NS1 co-binding region was performed. First, motif analysis of the co-localization regions of NF-κB and NS1 at a promoter region of less than 3k bp was performed. A total of 228 enriched terms for the binding motifs were obtained from the TRANSFAC database, with the E2F transcription factor and its family member, E2F-2, as the top two terms (**Figure 5A**). In addition, other E2 Factor (E2F) family member motifs, including E2F-1, were identified (**Supplementary Table. 4**).

Interestingly, a previous report revealed that the overexpression of NS1 attenuated the binding affinity of E2F-1 with the E2F motif region (Jiang et al., 2013). By analyzing the whole co-localization regions, we found significant enrichment of ETF (TEA domain family member 2) (p=1.248x10^-33^) and E2F-2 family members motifs (p=1.798x10^-32^) (**Figure 5B**). Together, our results indicate that NS1 dominated the binding approach of the transcription factors.

## Discussion

NF-κB, a master transcription factor, responds to viral infection and orchestrates the first line of antiviral gene expression to inhibit viral replication and transcription *via* RIG-I signaling (Hayden and Ghosh, 2011; Onomoto et al., 2021; Song and Li, 2021). One of the strategies of viral immune evasion mechanisms involves the inhibition of RIG-I and NF-κB signaling (Rückle et al., 2012; Deng et al., 2018). NS1 curbs host immune reaction by counteracting RIG-I signaling (Hale et al., 2008) *via* translocation into the nucleus and binding to chromatin annotated to the *IFNB1* gene to inhibit type I interferon response (Anastasina et al., 2016). NS1 can also directly suppress IKK-mediated NF-κB activation and thus attenuate NF-κB-induced antiviral gene expression (Gao et al., 2012). This study revealed the co-localization of NF-κB and NS1 using ChIP-seq. Further, the co-localization of NF-κB and NS1 at type III interferon genes, *IFNL1, IFNL2*, and *IFNL3*, was confirmed using the ChIP-reChIP assay. SEAP assay revealed the binding manners of NS1 on NF-κB binding sites. Further, the reporter assay showed the functions of NS1 and NF-κB at *IFNL1, IFNL2*, and *IFNL3* loci. Notably, we demonstrated that NS1 attenuated the expression of one of the type III interferon genes, *IFNL1*, by leveraging the displacement of NF-κB from its promoter and reducing the enrichment of RNA polymerase II at the promoter, leading to the downregulation of *IFNL1.* Besides, the reduction of H3K27ac enrichment at the *IFNL1* promoter was observed, aligning with the downregulation of *IFNL1* expression during IAV infection. Deletion of NS1 enhanced *IFNLs* expression; however, the block of NF-κB suppressed *IFNLs* expression, suggesting the competitive roles of NS1 and NF-κB in the mechanisms of type III interferon. Motif analysis of the NF-κB and NS1 co-localization regions revealed enriched E2F transcription factor binding motifs, suggesting the domination of NS1 in transcription factor-binding manners (Jiang et al., 2013). Indeed, the published RNA-seq data indicated that the deficiency of IAV in NS1 enhanced the *TLRs signaling pathway* and *RIG-I like receptor signaling pathway* and triggered substantial surges of *IFNB1* and *IFNLs* expression compared to wild type IAV infection (Eggenberger et al., 2019; Li et al., 2019).

We also reported type III IFNs gene with the different expression kinetics between two group cells were infected with IAV at different MOI (**Supplementary Figure 5**). At an MOI of 5, early upregulation of mRNA expression was observed as the time of addition high viral load infection increased to 6 h and then a more significant reduction was induced at 8 hpi. In contrast, a gradual accumulation of the type III IFNs transcripts was the characteristic of the A549 cells infected at MOI 0.5 (2-14 hpi). The observed repression of type III IFNs gene transcription at MOI 5 (6–8 hpi) compared with infection at MOI 0.5 may be indicative of host immune evasion due to increased virus burden, such as those identified in previous single-cell RNA-sequencing (scRNA-seq) studies that also identified the IAV infection dynamics of the viral defense and host innate immune response are caused by the interaction between cellular and viral heterogeneity (Ramos et al., 2019; Russell et al., 2019). At the same time, our data demonstrate that newly synthesized NS1 localized in the cytoplasm at early time of infection (2-4 hpi), shortly thereafter, NS1 proteins are mainly localized into the nucleus in the high MOI infected cells (93%) at 6 hpi and remained in the nucleus until at least 10 hpi (**Supplementary Figure 6**). These findings implicate high viral protein synthesis and nuclear translocation could initiate a feedback mechanism repressing the innate immune genes transcription in the A549 cells.

Type III IFNs were discovered in 2003 (Kotenko et al., 2003; Sheppard et al., 2003), serving as the latest recognition of the IFN family. Both human and mouse *IFNL2* and *IFNL3* genes are highly conserved and located near each other in the opposite direction, sharing highly similar promoters (Osterlund et al., 2007; Peterson et al., 2019). Although type I and III IFN promoters have binding sites for the same group of transcription factors, only interferon regulatory factors 3, 7 (IRF3, IRF7), and NF-κB are required for type III IFN expression. Thomson et al. reported that IRF3, 7, and NF-κB activate *IFNL1* gene expression independently *via* spatially separated promoter elements. Furthermore, the distal promoter region contains a cluster of NF-κB sites that are required for full induction of *IFNL1*. The depletion of the NF-κB p65 protein seriously reduces the level of *IFNL1* mRNA expression in LPS-stimulated dendritic cells (Odendall and Kagan, 2015). Additionally, inhibition of the NF-κB and IRF pathways revealed that type III IFN gene expression was more dependent on the NF-κB pathway than type I IFN gene expression, which was more dependent on the IRFs (Iversen et al., 2010). Thus, type III IFN regulation might be more flexible than type I IFN, and NF-κB plays a pivotal role in its activation. This finding is consistent with IAV NS1 protein-mediated suppression of type III IFN signaling *via* NF-κB recruitment interference, which was found in our study.

The retinoic-acid-inducible gene I (RIG-I) protein, one of the pattern recognition receptors (PRRs), plays an essential role in detecting IAV invasion. The activated RIG-I can induce the entrance of NF-κB into the nucleus, followed by induction of IFN genes, of which type III IFNs appear first, followed by type I IFNs, if necessary, to resist infection (Levy et al., 2011). NS1 can antagonize RIG-I or NF-κB in the cytoplasm to block the translocation of NF-κB into the nucleus, suggesting the role of NS1 in the inhibition of NF-κB signaling and curbing the expression of IFNλs (Hale et al., 2008). Deletion of NS1 promoted NF-κB signaling and interferon responses in our re-analysis of published data, consistent with the previous results (Wang et al., 2000); however, its mechanism is still not fully understood.

Overall, our study offers new insights into the mechanism underlying NS1 protein-mediated suppression of type III IFN signaling and a mechanistic explanation for the marked reduction in IFN-λ1 in critically ill patients with flu.

## Data availability statement

The ChIP-seq data presented in the study are publicly available at SRA Run Selector. We have released our data. The link is as follows: https://trace.ncbi.nlm.nih.gov/Traces/study/?acc=PRJNA857109&o=acc_s%3Aa (BioProject accession: PRJNA857109).

## Author contributions

Conceptualization, M-CL, C-PY and C-HH. Methodology, C-PY, M-TL and J-RY. Bioinformatics analyses, M-CL and M-TL. Validation, X-HC, J-RY and A-YC. Writing, M-CL and C-HH. Funding acquisition, M-CL and C-HH. All authors have read and agreed to the published version of the manuscript.

## Funding

This study was funded by MOST, Taiwan (MOST 107-2911-I-016-501; 108-2911-I-016-501 111-2321-B-016-002 to C-HH and 109-2311-B-016-001 to M-CL), and Ministry of National Defense Medical Affairs Bureau (MAB-108-077; MAB-109-037 to C-HH).

## Acknowledgments

We would like to thank Dr. Chuan-Chang Chuang (National Defense Medical Center, Taiwan) for providing the materials used for experiments.

## Conflict of interest

The authors declare that the research was conducted in the absence of any commercial or financial relationships that could be construed as a potential conflict of interest.

## Publisher’s note

All claims expressed in this article are solely those of the authors and do not necessarily represent those of their affiliated organizations, or those of the publisher, the editors and the reviewers. Any product that may be evaluated in this article, or claim that may be made by its manufacturer, is not guaranteed or endorsed by the publisher.
