# Supplementary material for: Influenza A virus NS1 protein represses antiviral immune response by hijacking NF-κB to mediate transcription of type III IFN
Source: Front Cell Infect Microbiol. 2022 Sep 15;12:998584. doi: 10.3389/fcimb.2022.998584 (PMC9519859; doi:10.3389/fcimb.2022.998584)
Supplement: Supplementary file 1 [file Presentation_1.pdf]

Supplementary file 1:

Supplementary Figure 1. Physical distributions of NF-κB and NS1 binding sites across genome.

Supplementary Figure 2. NS1 drove SEAP activity upon binding on NF-κB binding sites.

Supplementary Figure 3. RNA-seq analysis of IAV (wild type and ΔNS1) infected or non-infected cells.

Supplementary Figure 4. *IFNβ1* was regulated by NF-κB.

Supplementary Figure 5. Kinetics of *IFNLs* expression for the indicated time in the early stages of IAV infection

Supplementary Figure 6. NS1 was translocated and accumulated into the nucleus.

p65

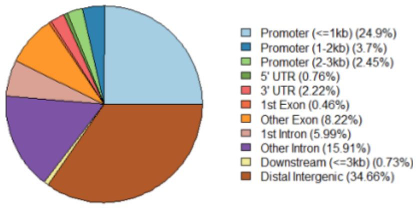

p50

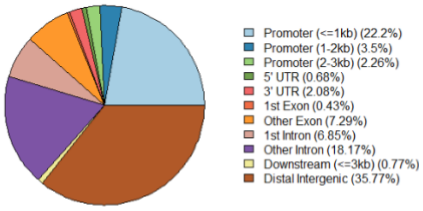

NS1

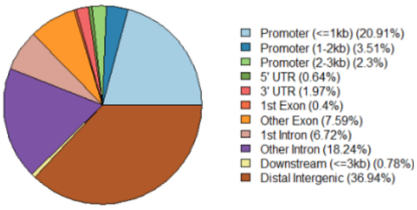

NF-κB and NS1 co-localization

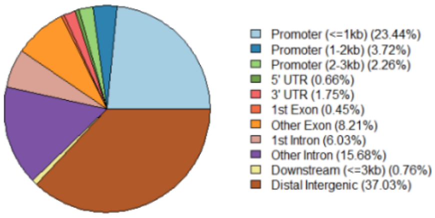

Supplementary Figure 1. Physical distributions of NF-κB and NS1 binding sites across genome. Pie chart showed percentages of physical distributions of NF-κB, NS1 and co-localization binding sites across genome.

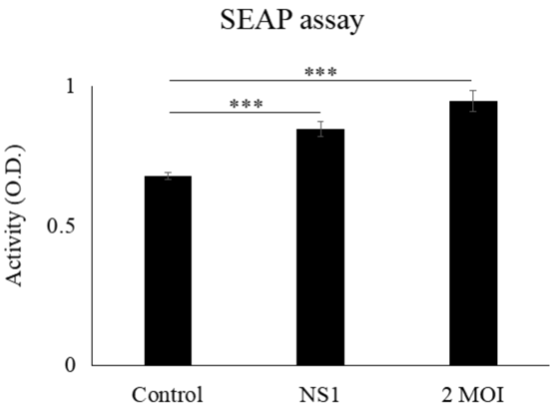

Supplementary Figure 2. NS1 drove SEAP activity upon binding on NF-κB binding sites.
A549 dual reporter cells (InvivoGen) were transfected with an empty vector control pCMV6 (OriGene, PS100001), the NS1 expression plasmid (OriGene, VC102470) or infected with WSN (MOI=2) for 24 hours before NF-κB–SEAP activity was determined by using QUANTI-Blue (InvivoGen). Biological triplicates of every group were performed to confirmed SEAP activity. Triple stars (***) represented p value < 0.001.

A

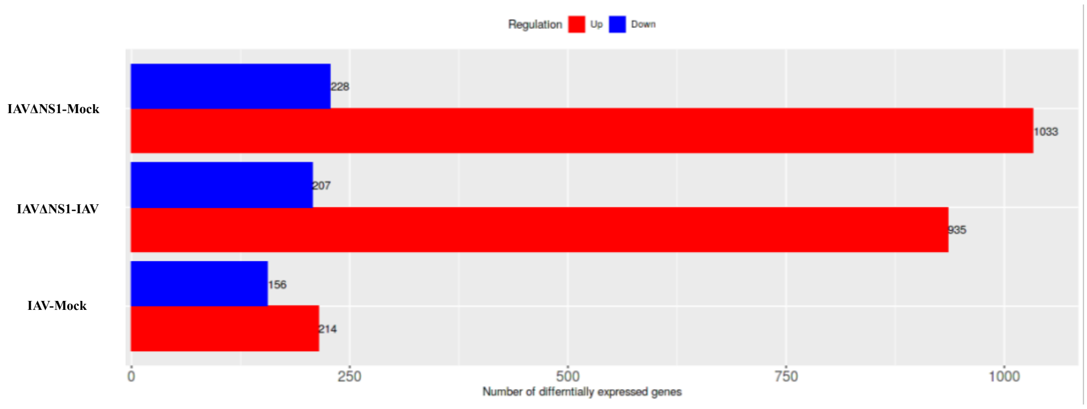
Regulation
Up
Down
IAVΔNS1-Mock
IAVΔNS1-IAV
IAV-Mock
228
1033
207
935
156
214
0
250
500
750
1000
Number of differentially expressed genes

B

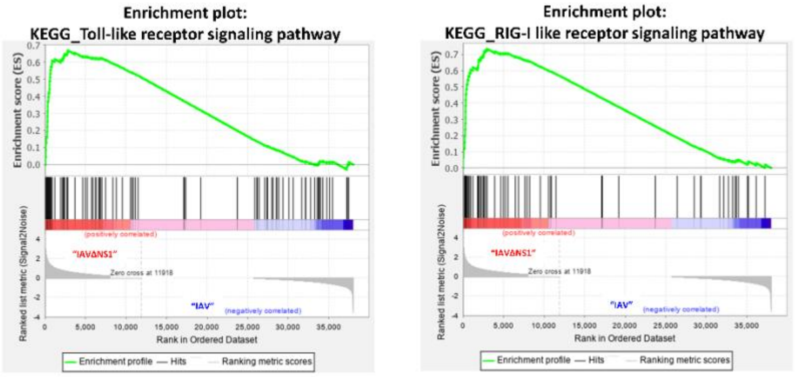
Enrichment plot:
KEGG_Toll-like receptor signaling pathway
Enrichment plot:
KEGG_RIG-I like receptor signaling pathway
Enrichment score (ES)
Ranked list metric (Signal2Noise)
(positively correlated)
"IAVΔNS1"
Zero cross at 11918
"IAV"
(negatively correlated)
Rank in Ordered Dataset
Enrichment profile
Hits
Ranking metric scores

C

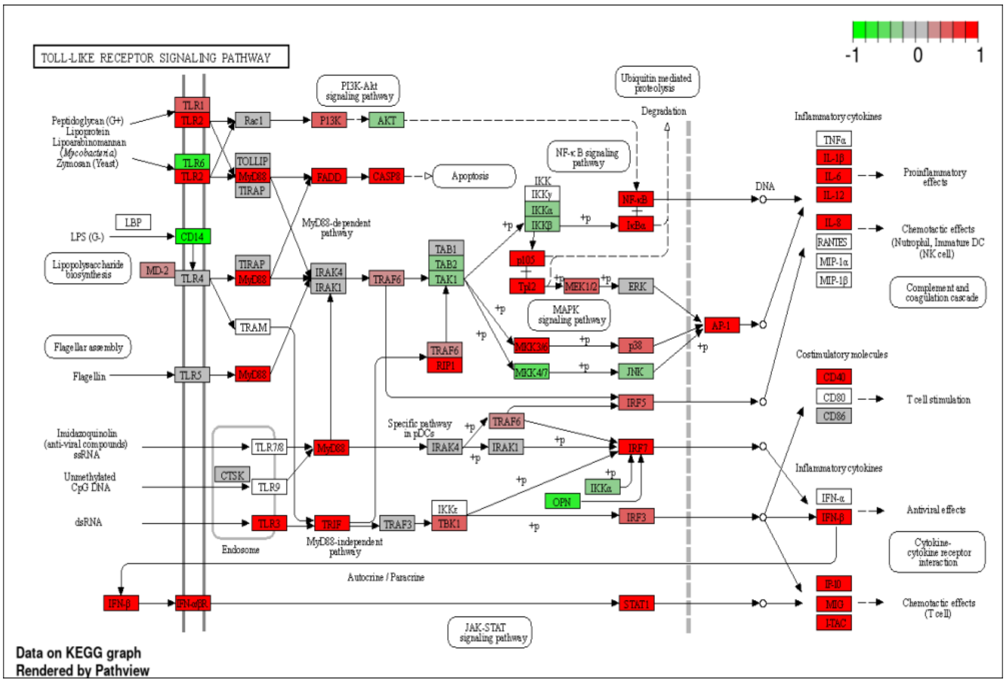
TOLL-LIKE RECEPTOR SIGNALING PATHWAY
-1
0
1
PI3K-Akt signaling pathway
Ubiquitin mediated proteolysis
Degradation
Inflammatory cytokines
Peptidoglycan (G+)
Lipoprotein
Lipoarabinomannan (Mycobacteria)
Zymosan (Yeast)
TLR1
TLR2
TLR6
Rac1
PI3K
AKT
TOLLIP
MyD88
TIRAP
FADD
CASP8
Apoptosis
NF-κB signaling pathway
IKK
IKKγ
IKKα
IKKβ
NF-κB
IκBα
DNA
TNFα
IL-1β
IL-6
IL-12
IL-8
RANTES
MIP-1α
MIP-1β
Proinflammatory effects
Chemotactic effects (Nutrophil, Immature DC (NK cell)
Complement and coagulation cascade
LBP
LPS (G-)
CD14
MyD88-dependent pathway
Lipopolysaccharide biosynthesis
MD-2
TLR4
TRAM
IRAK4
IRAK1
TRAF6
TAB1
TAB2
TAK1
p105
Tpl2
MEK1/2
ERK
MAPK signaling pathway
MKK3/6
p38
MKK4/7
JNK
AP-1
RIP1
Flagellar assembly
Flagellin
TLR5
Costimulatory molecules
CD40
CD80
CD86
T cell stimulation
IRF5
Imidazoquinolin (anti-viral compounds)
ssRNA
TLR7/8
Specific pathway in pDCs
IRF7
Unmethylated CpG DNA
CTSK
TLR9
OPN
Inflammatory cytokines
IFN-α
IFN-β
Antiviral effects
dsRNA
TLR3
TRIF
TRAF3
IKKε
TBK1
IRF3
Endosome
MyD88-independent pathway
Cytokine-cytokine receptor interaction
Autocrine / Paracrine
IFN-β
IFN-αβR
STAT1
IP-10
MIG
I-TAC
Chemotactic effects (T cell)
JAK-STAT signaling pathway
Data on KEGG graph
Rendered by Pathview

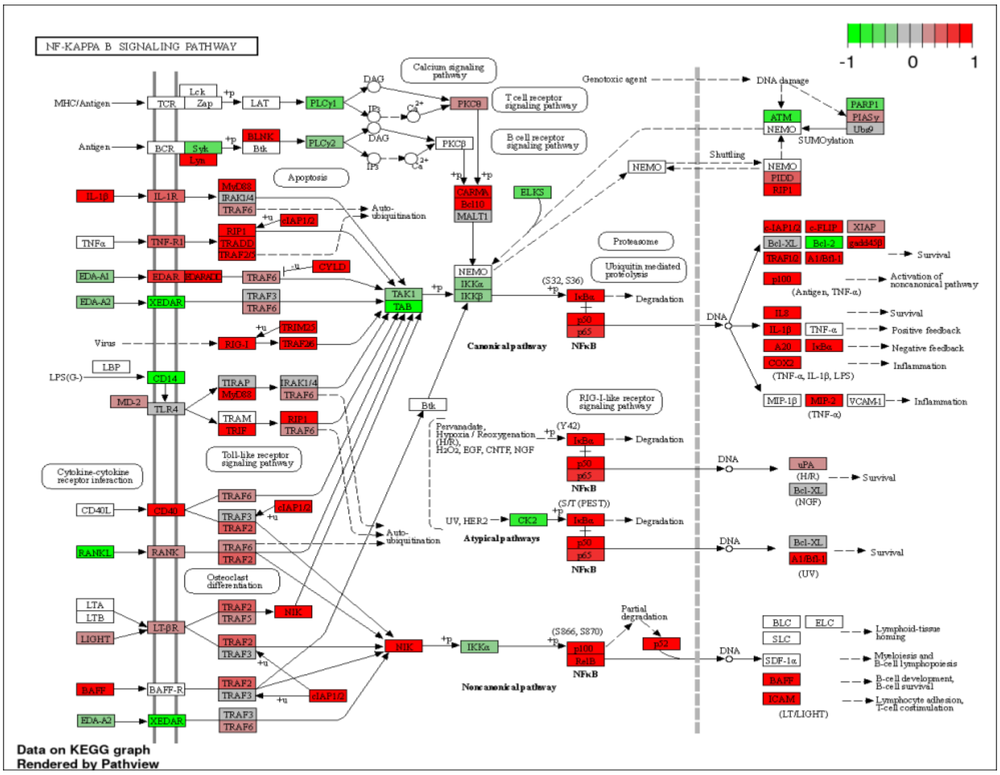

D

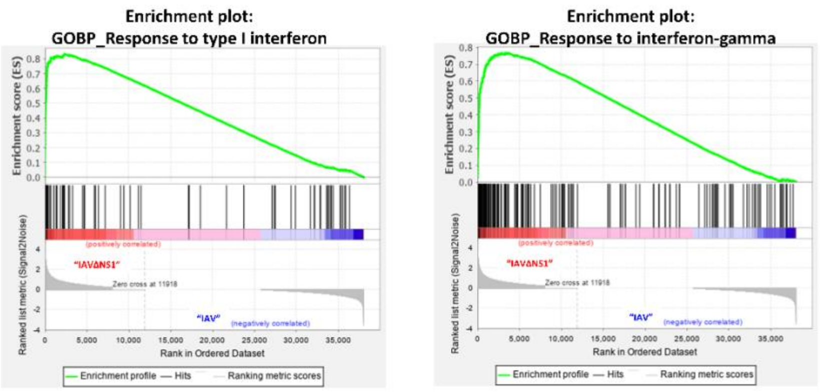

Supplementary Figure 3. RNA-seq analysis of the wild type and the deletion of NS1 IAV-infected or non-infected cells. A. Bar chart showed numbers of up and down-regulation of differentially expressed genes among mock, wild type, and the deletion of NS1 IAV (IAVΔNS1) groups. B. Enrichment plot of KEGG pathways analysis between the deletion of NS1 versus wild type IAV. C. Plot of KEGG graph between the deletion of NS1 versus wild type IAV. D. Enrichment plot of biological process analysis between the deletion of NS1 versus wild type IAV. RNA-seq data derived from GSE147507. GOBP, biological process of gene ontology.

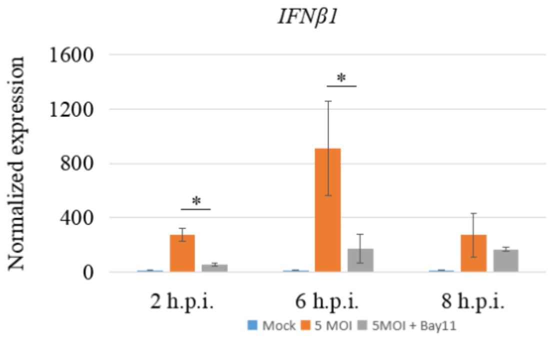

Supplementary Figure 4. ***IFNβ1*** **was regulated by NF-κB.**

A549 cells with or without pretreatment of NF-κB inhibitor Bay11(20 uM for 2 hours) were infected with or without WSN (MOI=5) for 2, 6 and 8 hours. Biological duplicates of every group were performed to confirmed relative expression of *IFNβ1/GAPDH*. One star (*) represented p value < 0.05.

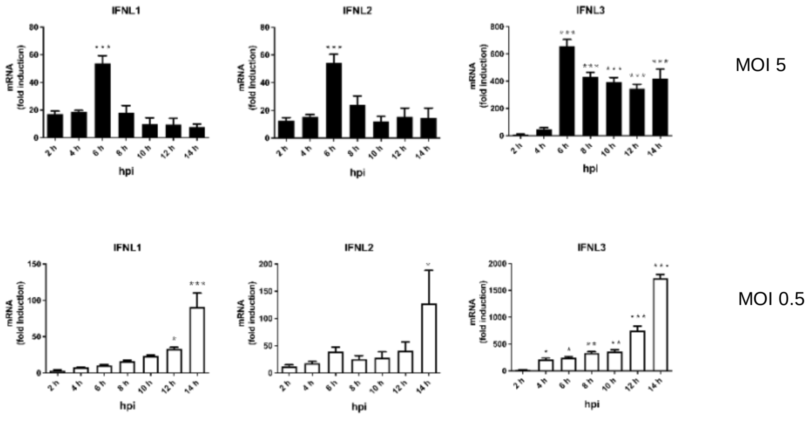

Supplementary Figure 5. Kinetics of *IFNLs* gene expression for the indicated time in the early stages of IAV infection.

A549 cells were infected with IAV either at indicated time (2, 4, 6, 8, 10, 12, and 14 hpi) at a MOI of 5 (upper) or 0.5 (lower), then the cells were harvested for further reverse transcription-quantitative PCR analysis of type III *IFNLs* expression. Data are presented as the mean ± SD of three individual experiments.

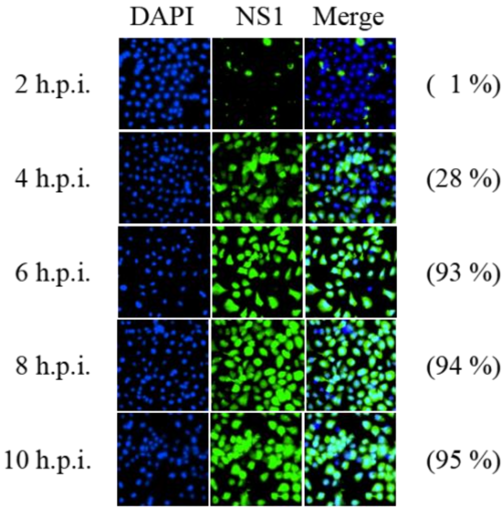

Supplementary Figure 6. NS1 was translocated and accumulated into the nucleus.
A549 cells were infected with WSN (H1N1) virus at an MOI of 5 for 2, 4, 6, 8 and10 hours, the localization of the NS1 protein was determined by immunofluorescence assay. Indirect immunofluorescence was carried out using a polyclonal rabbit anti-NS1 antibody against the NS1 protein (green) and DAPI staining for the nuclei (blue). The percentage of cells showing the nuclear localization of NS1 was scored from 100 cells.
