# Supplementary material for: Influenza A virus NS1 protein represses antiviral immune response by hijacking NF-κB to mediate transcription of type III IFN
Source: Front Cell Infect Microbiol. 2022 Sep 15;12:998584. doi: 10.3389/fcimb.2022.998584 (PMC9519859; doi:10.3389/fcimb.2022.998584)
Supplement: Supplementary file 2 [file Table_1.docx]

Supplementary Table 1. Primer pairs used for amplification of PCR.

| Gene name | Direction | Sequences | Usage |
| --- | --- | --- | --- |
| IFNL1 | Forward | CTA GAC CAG CCC CTT CAC AC | ChIP-reChIP PCR |
|  | Reverse | TCT TCC TGG GTC ACT CAC CT |  |
| IFNL2 | Forward | CCC CTC TAA CGC CCT CTA GT | ChIP-reChIP PCR |
|  | Reverse | TCC GCT AAG CAG GAC AGA TT |  |
| IFNL3 | Forward | ACC CGG GTC TGT CAT CTT C | ChIP-reChIP PCR |
|  | Reverse | CAT CCT TCC CCT GTG GTG |  |
| IFNL1 | Forward | CTA GAC CAG CCC CTT CAC AC | ChIP-qPCR |
|  | Reverse | TCT TCC TGG GTC ACT CAC CT |  |
|  | probe | /56-FAM/CAC CAC ATC /ZEN/CTC TCC CAG CT/3IABkFQ/ |  |
| IFNL2 | Forward | CCC CTC TAA CGC CCT CTA GT | ChIP-qPCR |
|  | Reverse | TCC GCT AAG CAG GAC AGA TT |  |
|  | probe | /56-FAM/CAG TCC TGC /ZEN/GCT CGG CTC CG/3IABkFQ/ |  |
| IFNL3 | Forward | ACC CGG GTC TGT CAT CTT C | ChIP-qPCR |
|  | Reverse | CAT CCT TCC CCT GTG GTG |  |
|  | probe | /56-FAM/CCT CCT GGA /ZEN/GCT GGT GCA GC/3IABkFQ/ |  |
| IFNB1 | Forward | GTCAGAGTGGAAATCCTAAG | RT-qPCR |
|  | Reverse | ACAGCATCTGCTGGTTGAAG |  |

Supplementary Table 2. Primers for plasmid construction

| **primer name** | **sequence** |
| --- | --- |
| IFNL1 (EcoR I) | CGGAATTCCGCTAGACCAGCCCCTTCACAC |
| IFNL1 (BamH I) | CGGGATCCCGTCTTCCTGGGTCACTCAC |
| IFNL2 (EcoR I) | CCGGAATTCCCCCCTCTAACGCCCTCTAGT |
| IFNL2 (XhoI) | CCGCTCGAGTCCGCTAAGCAGGACAGATT |
| IFNL3 (EcoR I) | CGGAATTCCGGCCTGGGTGACAGAGTAAGG |
| IFNL3 (BamH I) | CGGGATCCCGGAAATTCACCCAGTGAGAACG |

Supplementary Table 3. Enriched pathways in DEGs for the selected comparison:
